# Effects of a 10-Week Exercise and Nutritional Intervention with Variable Dietary Carbohydrates and Glycaemic Indices on Substrate Metabolism, Glycogen Storage, and Endurance Performance in Men: A Randomized Controlled Trial

**DOI:** 10.1186/s40798-024-00705-9

**Published:** 2024-04-10

**Authors:** Anna Maria Moitzi, Martin Krššák, Radka Klepochova, Christoph Triska, Robert Csapo, Daniel König

**Affiliations:** 1 Division of Nutrition, Exercise and Health, Department of Nutritional Sciences, Faculty of Life Sciences, University of Vienna, Vienna, Austria; 2 Vienna Doctoral School of Pharmaceutical, Nutritional and Sport Sciences, University of Vienna, Vienna, Austria; 3 Division of Nurtition, Exercise and Health, Department of Sport and Human Movement Science, University of Vienna, Vienna, Austria; 4 Department of Biomedical Imaging and Image Guided Therapy, High Field MR Centre of Excellence, Medical University of Vienna, Vienna, Austria; 5 Division of Endocrinology and Metabolism, Department of Internal Medicine III, Medical University of Vienna, Vienna, Austria; 6 Leistungssport Austria, High Performance Centre, Brunn am Gebirge, Lower Austria Austria; 7 Division of Training Science, Department of Sport and Human Movement Science, University of Vienna, Vienna, Austria

**Keywords:** Glycaemic index, High fat, Carbohydrates, Endurance performance, Muscle glycogen, Substrate metabolism, IMCL, Energy storage

## Abstract

**Background:**

Daily nutrition plays an important role in supporting training adaptions and endurance performance. The objective of this 10-week study was to investigate the consequences of varying carbohydrate consumption and the glycaemic index (GI) together with an endurance training regimen on substrate oxidation, muscle energy storage and endurance performance under free-living conditions. Sixty-five moderately trained healthy men (29 ± 4 years; VO_2_ peak 55 ± 8 mL min^−1^ kg^−1^) were randomized to one of three different nutritional regimes (LOW-GI: 50–60% CHO with ≥ 65% of these CHO with GI < 50 per day, n = 24; HIGH-GI: 50–60% CHO with ≥ 65% CHO with GI > 70 per day, n = 20; LCHF: ≤ 50 g CHO daily, n = 21). Metabolic alterations and performance were assessed at baseline (T0) and after 10 weeks (T10) during a graded exercise treadmill test. Additionally, a 5 km time trial on a 400-m outdoor track was performed and muscle glycogen was measured by magnet resonance spectroscopy.

**Results:**

Total fat oxidation expressed as area under the curve (AUC) during the graded exercise test increased in LCHF (1.3 ± 2.4 g min^−1^ × km h^−1^, *p* < 0.001), remained unchanged in LOW-GI (*p* > 0.05) and decreased in HIGH-GI (− 1.7 ± 1.5 g min^−1^ × km h^−1^, *p* < 0.001). After the intervention, LOW-GI (− 0.4 ± 0.5 mmol L^−1^ × km h^−1^, *p* < 0.001) and LCHF (− 0.8 ± 0.7 mmol L^−1^ × km h^−1^, *p* < 0.001) showed significantly lower AUC of blood lactate concentrations. Peak running speed increased in LOW-GI (T0: 4.3 ± 0.4 vs. T10: 4.5 ± 0.3 m s^−1^, *p* < 0.001) and HIGH-GI (T0: 4.4 ± 0.5 vs. T10: 4.6 ± 0.4 m s^−1^), while no improvement was observed in LCHF. Yet, time trial performance improved significantly in all groups. Muscle glycogen content increased for participants in HIGH-GI (T0: 97.3 ± 18.5 vs. T10: 144.5 ± 39.8 mmol L wet-tissue^−1^, *p* = 0.027) and remained unchanged in the LOW-GI and the LCHF group. At the last examination, muscle glycogen concentration was significantly higher in LOW-GI compared to LCHF (*p* = 0.014).

**Conclusion:**

Changes in fat oxidation were only present in LCHF, however, lower lactate concentrations in LOW-GI resulted in changes indicating an improved substrate metabolism. Compared to a LCHF diet, changes in peak running speed, and muscle glycogen stores were superior in LOW- and HIGH-GI diets. The low GI diet seems to have an influence on substrate metabolism without compromising performance at higher intensities, suggesting that a high-carbohydrate diet with a low GI is a viable alternative to a LCHF or a high GI diet.

*Trial registration*: Clinical Trials, NCT05241730. https://clinicaltrials.gov/study/NCT05241730. Registered 25 January 2021.

**Supplementary Information:**

The online version contains supplementary material available at 10.1186/s40798-024-00705-9.

## Key Points

- While only the LCHF diet showed an improvement in fat oxidation and the HIGH-GI group increased peak running speed and showed a trend towards an improvement in time to exhaustion, the LOW-GI group appears to combine both effects on substrate metabolism and performance.
- Administered under free-living conditions, energy intake changed in LOW-GI diet, possible due to higher intake of fibre, stable insulin levels and associated higher feeling of satiety. Energy intake in HIGH-GI showed no changes and in LCHF a trend towards a reduced intake was observed.
- While changes in body weight and composition were more pronounced in LOW-GI and LCHF group, muscle energy storage showed beneficial improvements in endurance runners on a high glycaemic index diet. With the muscle glycogen content being significantly lower in LCHF compared to LOW-GI group, it can be assumed that this might be a limiting factor in maximum values for the graded exercise test.

**Supplementary Information:**

The online version contains supplementary material available at 10.1186/s40798-024-00705-9.

## Background

Carbohydrates have been recognized as pivotal in maintaining performance during prolonged endurance exercise [1–5]. This crucial importance of maintaining an optimal carbohydrate supply for endurance athletes has been the subject of scientific discussion for more than a century [6, 7]. Full glycogen stores [8] combined with an optimal metabolic flexibility are key energetic components for high endurance performance [9]. Metabolic flexibility refers to the ability of an organism to adapt fuel oxidation to fuel availability [10]. Based on the availability of carbohydrates and fats, with an optimal metabolic flexibility the body can efficiently switch between those energy sources. During the transition from rest to exercise, energy requirements in the working muscle increase drastically, with duration, and intensity influencing substrate selection, i.e., whether carbohydrates or fats are used to produce ATP. As exercise intensity increases, carbohydrates become the preferred energy source because energy provision of fats does no longer provide sufficient energy per unit of time in the form of adenosine triphosphate (ATP) for muscle contractions [11–13]. Previous studies have shown convincingly that as intensity increases, fat oxidation decreases at the expense of carbohydrate oxidation [14, 15]. It can therefore be concluded that carbohydrates can play a decisive role, especially during intensive prolonged endurance exercise. Since storage capacity for carbohydrates in muscle and liver is limited to approximately 1500 to 2000 kcal [8, 16], endurance training aims to enhance fat oxidation and slow down carbohydrate oxidation so that intramuscular and intrahepatic glycogen stores are spared.

Daily nutritional intake represents an essential role in supporting metabolic adaptions through training. A very effective diet to date to achieve adjustments in metabolism is a low carbohydrate high fat (LCHF) diet. Long-term LCHF diets have been shown to increase maximal fat oxidation, both at rest as well as during submaximal exercise conditions [17–19]. However, what has also been observed is that possible but not exclusively due to the decreased activation of glycolytic enzymes (i.e. glycogen phosphorylase, phosphofructokinase, pyruvate dehydrogenase) during a LCHF diet, only a very small positive effect or no effect on VO_2_ max, a biomarker for endurance capacity, could be measured [19]. Regarding the respiratory exchange ratio (RER) and time to exhaustion (TTE) Cao et al. [20] found clear positive effects on RER, but for TTE the LCHF diet showed no benefits, even when empty glycogen stores were replenished shortly before competition [21]. As a result, performance at higher intensities is possibly limited after longer periods on high-fat diets on the one hand because of reduced glycogen stores [22] and on the other hand because of the mitigated carbohydrate metabolism [23]. In addition to this, other side effects such as decreased training capacity [24] or exercise economy [25] and reduced well-being, fatigue, gastrointestinal complaints, or poor concentration due to lack of micronutrients and glycogen can occur during or after the change to a LCHF diet [26].

To avoid side effects and possible performance losses of reduced daily carbohydrate intake, a high-carbohydrate diet with a low glycaemic index could be a promising approach [27]. The glycaemic index (GI) reflects the insulinemic response of a carbohydrate [28] and might therefore be able to influence substrate metabolism [29]. Insulin has been shown to inhibit fat oxidation and promote glucose oxidation via various mechanisms, so low GI foods (GI ≤ 55) result in a lower postprandial glucose and insulin response and thus a mitigated inhibition of fat oxidation compared to a high GI food [27]. There are already studies showing supporting results at present [30, 31]. In a recent publication, it was shown that although the influence on fat oxidation after a carbohydrate rich low GI diet was not as evident after a LCHF diet, low GI nutrition resulted in improved metabolic flexibility [31] and therefore higher performance improvements in an incremental cycling test [30]. In addition to that, the low GI diet was more feasible and tolerated than the other nutritional regimes.

Long-term studies that examine the influence of a low GI diet compared to a LCHF diet on metabolic flexibility and performance outcomes are scarce. Therefore, the aim of this 10-week interventional study was to investigate the effects of a LCHF diet, a carbohydrate rich low GI diet (LOW-GI) and a carbohydrate rich high GI diet (HIGH-GI) on metabolic parameters, peak running speed (PRS), running economy (RE) and peak oxygen consumption in a graded exercise test, performance in a 5-km time trial (TT) and muscle energy stores. We wanted to test the hypothesis whether a high carbohydrate low GI diet is able to influence fat oxidation to a similar extent as a LCHF diet without restricting carbohydrate oxidation. Furthermore, we hypothesise that the LOW-GI group will experience similar improvements in PRS and TT as the HIGH-GI group. As for muscle glycogen stores, we expect them to decrease in the LCHF diet, whereas no noticeable difference is expected in the LOW-GI and HIGH-GI groups.

## Methods

### Study Design

**Fig. 1:**
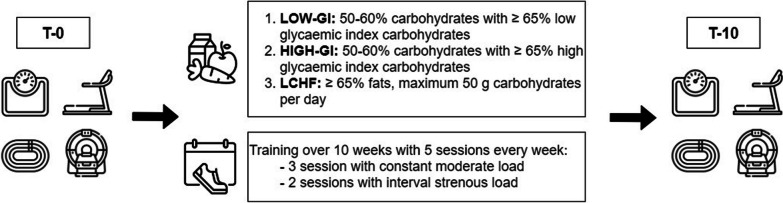
Overview of the study schedule. Examinations at baseline (T-0) and after the intervention (T-10): bioelectrical impedance analysis, graded exercise test on treadmill, 5 km time trial on track, and magnetic resonance scan

The present study was adopted a controlled, open, randomised, non-blinded study. The recruitment of participants was carried out through flyers, social media, and the university sports centre. The study was approved by the Ethical Committee of the Medical University of Vienna (EK Nr: 2105/2021), the Ethical Committee of the University of Vienna (Reference Number: 00871), registered at Clinical Trials (NCT05241730) and conducted in accordance with the Declaration of Helsinki. The study lasted 10 weeks and combined a prescribed endurance training with a nutritional intervention. A comprehensive overview of the study schedule can be seen in Fig. 1.

### Participants

A total of 121 males registered for the trial. After an initial telephone screening, where the study objectives were explained in detail and inclusion criteria (age, training sessions per week, and physical activity readiness) were checked, 87 men were invited for an initial medical examination, including a medical history questionnaire to ensure the inclusion criteria were met and that there was no medical condition that could be worsened by the study protocol.

Inclusion criteria included age ranging from 18 to 40 years and 2–3 training sessions per week (no professional level athlete) as well as the evaluation of readiness for physical activity (PAR-Q). Exclusion criteria were previous experience with one of the intervention diets, being a professional athlete, contraindications to physical activity according to the American College of Sports Medicine Guidelines diagnosed by a medical doctor [32], use of medications or dietary supplements that could affect measurements or are prohibited by the WADA Code, chronic diseases, and arterial hypertension. Additionally, screening for magnetic resonance spectrometry specific exclusion criteria was performed, which included claustrophobia, pacemaker, cochlear implant, subcutaneous injection system, stents, metal implants, piercings, and tattoos, or a body weight of more than 180 kg.

In total, 87 healthy recreationally active endurance athletes met all the criteria, provided their written informed consent, and were enrolled in the study. Regarding the sample size, previous studies with a comparable design [30, 31] as well as an a priori power analysis with G*power (version 3.1.9.7. for windows) were used [33]. An estimated partial eta square of 0.025 with a power of 0.8, α error probability of 0.05 and a surcharge for dropouts resulted in a sample size of 26 subjects per group, which was thought to be sufficient to reach significant results and adequate power. To achieve a power of 80% and reliable statistical analyses, 20 subjects per group must complete the study under the specified conditions.

### Measurements

During the first visit, anthropometric and demographic data, such as body composition measured by bioelectric impedance (Seca mBCA 514/515, seca GmbH & co. KG, Hamburg, Germany) and training status, were collected. At a second visit, a graded exercise test starting at 6 km h^−1^ with increasing speed by 1.5 km h^−1^ every three minutes until exhaustion was performed on a treadmill (Quasar med, h/p/cosmos sports & medical GmbH, Nussdorf-Traunstein, Germany). Capillary blood samples were collected and analysed using the Biosen lactate analyser (Biosen S-line, KF-diagnostic GmbH, Barleben/Magdeburg, Germany) at baseline, at the end of each step during the test and at exhaustion. The blood lactate thresholds (LT and individual anaerobic threshold IAT) were furthermore calculated from the blood lactate curve using Ergonizer software (Freiburg, Germany) in order to define the training zones for the prescribed training plan. LT is thereby defined as the earliest moment of an increase in blood lactate concentration with increasing exercise intensity. Moreover, Ergonizer software calculated the IAT according to the principle of the net increase via the lactate concentration at the LT. This means that the IAT is defined as the point that represents the LT plus 1.5 mmol L^−1^. If the final increment could not be completed, the peak running speed (PRS) was calculated as proposed by [34]:

$$PRS=V \, com+ \frac{t}{3}* \Delta V$$

in which V com is the last increment completed, t the number of minutes the final not completed speed was sustained, and ∆V the final speed increment (1.5 km h^−1^). The duration of the graded exercise test was calculated as the total duration of the test from the first increment (6 km h^−1^) on and is referred to as time to exhaustion (TTE).

During the test, oxygen uptake (VO_2_) and respiratory exchange ratio (RER) were continuously measured by a breath-by-breath gas analyser (MetaLyzer 3B, Cortex Biophysik GmbH, Leipzig, Germany) to determine peak VO_2_, which was defined as the 30 s rolling average maximum VO_2_ value during the graded exercise test. Before each experimental session, the device was calibrated using procedures according to the manufacturer’s instructions. The average values of VO_2_ (L min^−1^) and RER were calculated in the last minute of each 3-min exercise stage. Based on their peak oxygen uptake, subjects were randomly assigned to one of three dietary groups to minimise baseline differences, as suggested by Hopkins [35]. Allocation to an intervention group was random, but was monitored to ensure that peak VO_2_ values at the intervention were comparable in all three groups. Based on the average values of VO_2_ and RER, fat and carbohydrate oxidation were calculated using stochiometric equations up to RER = 1.00 according to Péronnet and Massicotte [36], where:

$${\text{VCO}}2 [{\text{L}} \, {{\text{min}}}^{-1}]=RER*VO2 [{\text{L}} \, {{\text{min}}}^{-1}]$$

$$Fat \, oxidation \left[{\text{g}} \, {{\text{min}}}^{-1}\right]=1.695*VO2 \left[{\text{L}} \, {{\text{min}}}^{-1}\right]-1.701* {\text{VCO}}2 [{\text{L}} \, {{\text{min}}}^{-1}]$$

$$Carbohydrate \, oxidation \left[\mathrm{g } \, {{\text{min}}}^{-1}\right]=4.21*{\text{VCO}}2 [\mathrm{L } \, {{\text{min}}}^{-1}]-2.962* VO2 \left[{\text{L}} \, {{\text{min}}}^{-1}\right]$$

To assess and compare the parameters during the graded exercise test, for fat and carbohydrate oxidation, RER and blood lactate the area under the curve (AUC) was calculated between the start of the test and the final increment completed by all participants before exhaustion (t_n_) using the following equation:

$$AU{C}_{0-{t}_{n}}=\frac{1}{2}\sum_{i=0}^{{t}_{n}}\left({t}_{i+1}-{t}_{i}\right)x\left({C}_{i}+{C}_{i+1}\right)$$

where t_n_ is the final completed increment, t_i_ the km h^−1^ of the increment at which the parameter was measured and C_i_ the respective concentration/value to that timepoint. Due to the fact that the number of completed increments developed differently in the groups during the study, the AUC of lactate concentration was related to the time to exhaustion (TTE) in the graded exercise test. For this purpose, the corresponding AUC was divided by the TTE and multiplied by 100.

Additionally, running economy at the lactate threshold was calculated for each subject. Therefore, the VO_2_ consumption in mL min^−1^ at lactate threshold was divided by body weight. Relative VO_2_ consumption and velocity at lactate threshold were used to calculate running economy in mL min^−1^ km^−1^, as proposed by [37].

In a third visit, a 5-km time trial (TT) on an outdoor 400 m track was performed. Body composition analyses, graded exercise test, as well as time trial were performed at the beginning and after the 10-week intervention under the same conditions and at the same time of day to minimise circadian influences. Body composition was measured in fasted state for all timepoints. For the time trial and the graded exercise test at the beginning all subjects were given a standardized nutritional guideline, for the 24 h prior to the test. They were instructed to fast and to restrain from caffeine 2 h prior to performing the test. Additionally, 48 h prior to the test intense exercise was not allowed. For the graded exercise test and the time trial at the end of the intervention nutrition was not controlled because of the different nutritional regimes that they followed. However, subjects were advised to fuel as they would fuel before a hard interval session and the last meal prior the test was eaten at the same timepoint like at the first examination. Again, no intense exercise took place 48 h prior to the tests.

In addition to performance tests, 24 (8 from each group) randomly selected subjects underwent magnetic resonance spectroscopy (MRS) of the vastus lateralis (VL) muscle at baseline and after the intervention to determine muscle glycogen and intramyocellular lipid (IMCL) content. After an overnight fast, measurements were performed in a 7 T whole-body magnetic resonance system (Magnetom, Siemens Healthcare, Erlangen, Germany). Spectroscopy measurements and calculations were done according to previous studies [38, 39]. In more detail, ^13^C MRS for the assessment of tissue glycogen was performed with ^1^H/^13^C body surface coil (STARK CONTRAST MRI coils Research, Erlangen, Germany) consisting of a slightly curved 18 cm transmitter loop with two smaller (14 cm) receiver elements combined with quadrature detection operating at ^13^C resonance frequency (74.73 MHz) and a 16 cm Tx/Rx loop for ^1^H imaging and shimming (297.2 MHz) applying 13^C^ pulse-acquire sequence (pulse length = 500 µs, flip angle = 90° in coil plane, repetition time = 500 ms, spectral bandwith = 10,000 Hz, number of acquisitions: 3 blocks of 512). Absolute glycogen concentrations were quantified by comparing the C1 glycogen doublet (100.5 ppm) integral of muscle spectra with that of a glycogen solution standard taken under identical conditions. Corrections for loading of the coil, coverage of sensitive volume of the coil on the muscle and subcutaneous adipose tissue thickness were performed. Intramyocellular lipids were quantified after repositioning the volunteers using a 28-channel knee coil (QED, Mayfield Village, OH, USA) by single voxel ^1^H MRS. T1 weighted, multi-slice localizer images were acquired and used for volume-of-interest (VOI) positioning. Spatial selection was achieved using a STEAM localization sequence and the VOI 15 × 15 × 30 mm^3^ was carefully placed in the VL muscle. Voxel positioning was guided by images to properly adjust voxel geometry and orientation to fit the actual shape of subject’s muscle, avoiding the boundaries of muscle and subcutaneous fat. Localized shimming was performed automatically followed by manual inspection and readjustments. The final linewidth of the water signal was in the range of 28–38 Hz in the magnitude mode. Signals of IMCL were measured with the following parameters: repetition time (TR)/echo time (TE) = 2000/20 ms; spectral bandwidth = 3 kHz; number of averages (NA) = 16. For acquisition of a concentration reference, the water signal was measured with a TR = 2000 ms and a TE = 20 ms; NA = 1.

### Nutrition Intervention

Under the supervision of trained dieticians, participants were instructed to follow the dietary pattern according to their respective group and previous pilot studies [30, 31]:

- LOW-GI: 50–60% carbohydrates with ≥ 65% of energy from low glycaemic index (GI < 50) carbohydrates per day
- HIGH-GI: 50–60% carbohydrates with ≥ 65% of energy from high glycaemic index (GI > 70) carbohydrates per day
- LCHF: ≥ 65% fat, maximum of 50 g carbohydrates per day

The dietary requirements were integrated into the test person's daily routine, and the meals were selected and prepared by the test person themselves. Food specifications for the LOW-GI group included among other things wholemeal bread, wild rice, vegetables other than potatoes or wholemeal pasta combined with an adequate protein (around 15% of total energy intake) and fat (around 30% of total energy intake) intake. For the HIGH-GI group, macronutrient distribution was comparable to the LOW-GI group with the advice to preferably consume white bread, pasta, rice, fruits, and potatoes along with other high GI foods. For the LCHF group the restrictions were the most drastic. For example, cereal products, carbohydrate sources, such as bread, pasta and rice, and sweet fruits had to be omitted and vegetable consumption was restricted to low-starch vegetables such as cabbage, cucumbers, and peppers. Only meat, fish and eggs were not restricted. To measure ketosis, the subjects in LCHF group carried out urine analyses with reagent strips (Ketostix, Acensia Diabetes Care Austria GmbH, Vienna, Austria) during the study. In addition, the level of ketone bodies in the capillary blood was measured with a ketone meter (On Call GK Dual, Acon Laboratories Inc., San Diego, California, USA) at the end of the intervention in fasted state. Additional file 1 showing the guidelines and example meals for each group was provided to the participants (see Additional file 1). All diets were ad libitum diets and subjects were advised not to restrict their energy intake. If they stuck to their dietary requirements, the test subjects were allowed to eat until they were full. In the event of concerns or questions about the nutritional regime, dieticians were contacted. Before starting the study, participants were asked to complete a 24-h recall and a food frequency questionnaire (FFQ). The validated DEGS1-FFQ was used and collects the frequency and quantity of 53 food items eaten in the last 4 weeks [40]. The questionnaire was completed online and converted to nutritional intake according to previous proposed methods [41]. 24-h recall and FFQ were combined to assess nutrition prior to the intervention. During the study, participants were asked to protocol one weekday and one weekend day per week. The protocols were reviewed by trained dieticians using nut.s software (Dato Denkwerkzeuge, Wien, Austria). The mean of 20 24-h recalls per subject was calculated to assess compliance during the study and used for further calculations.

Determination of the GI of the consumed foods was based on Atkinson, Foster-Powell [42] and Atkinson, Brand-Miller [43]. To calculate the GI of a meal, the amount of carbohydrates in grams per meal was determined. The GI of the individual foods in the meal was then calculated proportionately and added together. Thus, a GI was determined for each meal of the day. These were added and divided by the number of meals. Finally, the average GI of all protocols was determined for each subject.

### Exercise Intervention

The endurance training plan was the same for all groups and the training zones were customised for each subject based on the lactate thresholds resulting from the graded exercise test. An example week can be found in the Additional file 1. The plan consisted of five running sessions per week, three of which were steady-state and two of which were interval sessions. The steady-state runs were completed at the specified heart rate zones (below, at LT or between LT and IAT). For the intervals, the participants were guided by the pace specifications according to the prescribed zone (at or above IAT). Training sessions were distributed independently over the week, but long runs and the interval sessions were not performed on consecutive days. Additionally, there was be at least one rest day between the two interval sessions. During the first 4 weeks of the training plan, the focus was on training basic endurance, whereas in the second 4 weeks the focus was on interval sessions. The last 2 weeks were for maintenance. Basically, participants were encouraged to complete all training sessions. To be able to control this, all training sessions were recorded with a sports watch (Polar Vantage M, Polar Electro Oy, Kempele, Finland) and a heart rate belt (Polar H10, Polar Electro Oy, Kempele, Finland) and checked weekly by the study management. Nevertheless, it was established and communicated prior to commencement that a minimum requirement of 75% of the prescribed training minutes was necessary for inclusion in the final analysis.

Throughout the intervention phase, general condition, gastrointestinal well-being, and perceived effort were assessed daily using a visual analogue scale (VAS). This VAS is an assessment tool that quantitatively measures conditions on a scale ranging from 0 (optimal condition) to 100 (worst condition). The distance between the two conditions was marked by the participant and given in mm by the VAS [44].

### Statistical Analyses

Statistical analyses were performed using Statistical Package for the Social Sciences Software (SPSS for Windows, Version 28, SPSS Inc., Chicago, IL) and figures were created using GraphPad Prism (GraphPad Prism Version 8.0.2 for Windows, GraphPad Software, San Diego, California, USA). The level of significance was set at α = 0.05. The results of the descriptive analyses are shown as mean ± standard deviation (SD).

The normal distribution of the metric variables was tested by the Shapiro Wilk test. Continuous data without normal distribution, which cannot be transformed to normal distribution, were tested by a nonparametric method. Differences between groups at baseline were tested by one-way ANOVA. If normal distribution was not given, Kruskal–Wallis test was used. The Tukey post hoc test was performed to identify groups that differed significantly. For the assessment of the time (within subject factor), group (between subject factor) and time x group interaction effects, a two-way mixed ANOVA with Tukey-corrected post hoc analyses was used. In case of a significant group x time interaction, simple main effects for group and time were analysed. To compare the rate of changes from pre-intervention to post-intervention, the differences were calculated (post–pre) and further examined by one-way ANOVA. The effect sizes for one-way ANOVA (ηp^2^) and simple time effects (Cohen’s d) are displayed for significant results.

## Results

### Study Population

**Fig. 2:**
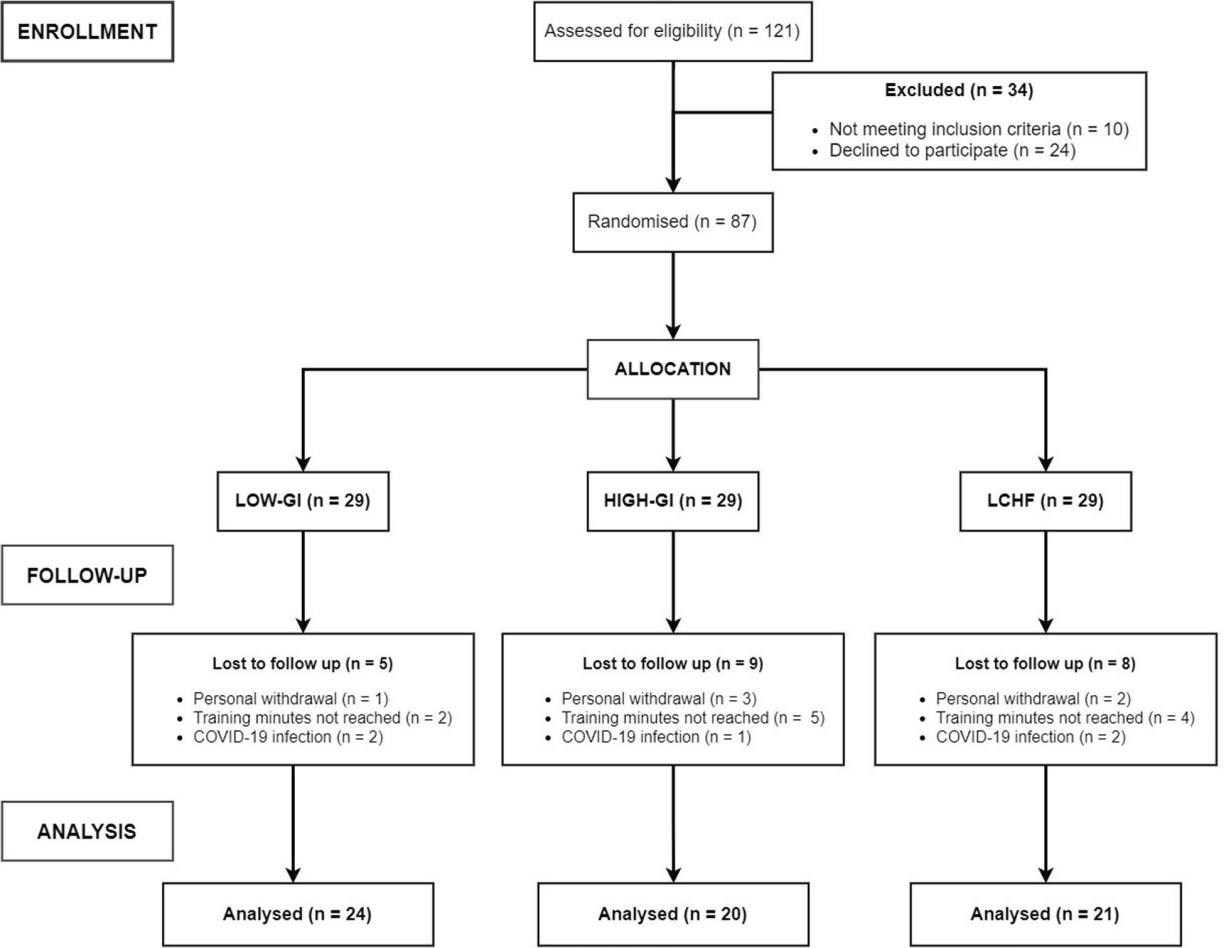
Flow chart of subject recruitment, randomisation, and follow-up

In total, 65 of the initial 87 randomised participants completed the trial and were included in the statistical analysis (24 subjects in the LOW-GI group, 20 subjects in the HIGH-GI group, and 21 subjects in the LCHF group). The reasons for the early withdrawal from the study can be depicted from Fig. 2. The reasons were not attributable to any adverse event during the examinations conducted or during the study, nor to difficulties triggered by the intervention. In addition, subjects were excluded from the analysis if the specified 75% of exercise minutes were not achieved.

The baseline characteristics are shown in Table 1. No baseline differences were found for age, height, weight, BMI, active days per week, or VO_2_ peak. Subjects were active on average of 3 days per week and had an average maximum oxygen uptake of 55 ± 8 mL min^−1^ kg^−1^.

**Table 1:** Baseline characteristics

	LOW-GI	HIGH-GI	LCHF	*p*-Value
Age [years]	30 ± 4	29 ± 4	27 ± 4	0.112
Height [cm]	181.5 ± 6.5	180.2 ± 5.6	182.0 ± 6.9	0.740
Weight [kg]	79.5 ± 8.1	77.1 ± 11.3	81.5 ± 10.8	0.354
BMI [kg m^−2^]	24.1 ± 2.8	23.7 ± 3.2	24.6 ± 3.3	0.661
Active days per week	3 ± 1	3 ± 1	3 ± 1	0.886
VO_2_ peak [mL min^−1^ kg^−1^]	54 ± 7	55 ± 7	55 ± 9	0.967

### Nutritional Intervention

Energy intake and macronutrient intake before and during the intervention are shown in Table 2. Before the intervention, no significant differences were observed in total energy intake or macronutrient intake (*p* > 0.050, respectively). Furthermore, the glycaemic index of the supplied was not different between the two carbohydrate groups (*p* > 0.050).

**Table 2:** Energy and macronutrient intake before (T-0) and during the intervention (T-10)

Group	T-0	T-10	Time x group	Time effect	Group effect during intervention period
Energy intake [kcal]					
LOW-GI	2178 ± 556	1784 ± 502	**0.006**	** < 0.001**	**0.028**
HIGH-GI	2072 ± 585	2124 ± 462^c^		0.550	
LCHF	1954 ± 657	1755 ± 468^b^		0.053	
Carbohydrate intake [%]					
LOW-GI	50.2 ± 5.5	50.5 ± 5.4^c^	** < 0.001**	0.839	** < 0.001**
HIGH-GI	50.2 ± 5.9	53.5 ± 5.6^c^		**0.005**	
LCHF	45.4 ± 10.4	10.6 ± 3.7^a,b^		** < 0.001**	
Carbohydrate intake [g]					
LOW-GI	267.7 ± 79.7	211.8 ± 57.7^b,c^	** < 0.001**	** < 0.001**	** < 0.001**
HIGH-GI	247.0 ± 74.0	269.1 ± 51.6^a,c^		**0.048**	
LCHF	219.8 ± 91.0	41.1 ± 13.2^a,b^		** < 0.001**	
Protein intake [%]					
LOW-GI	16.4 ± 3.1	19.9 ± 2.7^b.c^	** < 0.001**	** < 0.001**	** < 0.001**
HIGH-GI	16.8 ± 3.6	15.7 ± 2.7^a.c^		**0.042**	
LCHF	18.8 ± 4.0	28.1 ± 3.0^a.b^		** < 0.001**	
Protein intake [g]					
LOW-GI	87.1 ± 30.9	86.7 ± 28.1^c^	** < 0.001**	** < 0.001**	** < 0.001**
HIGH-GI	84.5 ± 32.9	81.4 ± 25.8^c^		**0.042**	
LCHF	88.3 ± 35.3	117.5 ± 35.1^a,b^		** < 0.001**	
Fat intake [%]					
LOW-GI	31.8 ± 5.9	28.6 ± 5.8^c^	** < 0.001**	**0.034**	** < 0.001**
HIGH-GI	30.7 ± 5.5	28.7 ± 4.6^c^		0.104	
LCHF	34.3 ± 8.8	62.2 ± 4.8^a.b^		** < 0.001**	
Fat intake [g]					
LOW-GI	74.3 ± 21.8	58.0 ± 23.9^c^	** < 0.001**	**0.004**	** < 0.001**
HIGH-GI	71.5 ± 28.5	68.8 ± 24.0^c^		0.561	
LCHF	72 ± 29.4	120.2 ± 35.5^a.b^		** < 0.001**	
Glycaemic index					
LOW-GI	62 ± 10	41 ± 3		** < 0.001**	** < 0.001**
HIGH-GI	57 ± 8	64 ± 3		** < 0.001**	

^a,b,c^Significantly different intake between groups during the intervention: ^a^compared to LOW-GI, ^b^compared to HIGH-GI, ^c^compared to LCHF. Bold numbers respresent a significant interaction effect or a significant simple main effect

For nutrition, the two-way mixed ANOVA revealed significant interaction effects for energy and macronutrient intake (*p* < 0.050, respectively). Total energy intake was significantly reduced in the LOW-GI group (− 394 ± 491 kcal, *p* < 0.001, d = 0.802), while no significant differences were found in the other two groups. The energy intake during the study differed significantly between the HIGH-GI (2124 ± 462 kcal) and the LCHF group (1755 ± 468 kcal) with a higher energy intake in the HIGH-GI group (*p* = 0.043, ηp^2^ = 0.109). Changes in energy intake due to the study were significantly different between the LOW-GI (− 394 ± 491 kcal) and the HIGH-GI group (+ 52 ± 379 kcal, *p* = 0.005, ηp^2^ = 0.112), while no difference in change was observed between LOW-GI and LCHF group (− 199 ± 444 kcal, *p* = 0.118). Relative carbohydrate intake increased during the study in the HIGH-GI group (+ 3.3 ± 4.7%, *p* = 0.005, d = 0.711), decreased in the LCHF group (− 34.8 ± 10.0%, *p* < 0.001, d = 3.503) and remained unchanged in the LOW-GI group (+ 0.3 ± 6.7%, *p* = 0.839). The relative carbohydrate intake during the study differed significantly between LCHF and the two carbohydrate groups (*p* < 0.001, ηp^2^ = 0.941). The relative protein intake changed significantly in all groups due to the intervention (*p* < 0.050 for all groups) and differed significantly during the study (for all pairwise comparisons *p* < 0.050, ηp^2^ = 0.773). For LOW-GI (+ 3.4 ± 2.4%, *p* < 0.001, d = 1.452) and LCHF (+ 9.3 ± 4.7%, *p* < 0.001, d = 1.975) protein intake increased significantly. On the contrary, protein intake decreased in the HIGH-GI group (− 1.1 ± 2.3%, *p* = 0.042, d = 0.487). Relative fat intake was significantly reduced in LOW-GI group (− 3.1 ± 6.9%, *p* = 0.034, d = 0.461), remained unchanged in the HIGH-GI group (− 2.0 ± 5.2, *p* = 0.104) and increased significantly in the LCHF group (+ 27.9 ± 8.9%, *p* < 0.001, d = 3.152). During the study, fat intake differed significantly between the LCHF group and the two carbohydrate groups (*p* < 0.001, ηp^2^ = 0.906).

In both carbohydrate groups, the glycaemic index changed significantly during the study. In the LOW-GI group, the glycaemic index was reduced from 62 ± 10 before the intervention to 41 ± 3 during the intervention (*p* < 0.001, d = 1.469). For the HIGH-GI group, the glycaemic index increased from 57 ± 8 to 64 ± 3 (*p* < 0.001, d = 0.993). The glycaemic index differed significantly between the groups (*p* < 0.001, d = 7.555). Compliance to the LCHF diet was good. Fasted ketone bodies concentration measured at the end of the intervention was 0.8 ± 0.4 mmol L^−1^.

### Exercise Intervention

No differences in training minutes were found neither in total (LOW-GI: 2125 ± 294 min, HIGH-GI: 2072 ± 285 min, LCHF: 2101 ± 255 min, *p* = 0.824), nor when splitting the minutes in steady-state (LOW-GI: 1468 ± 196 min, HIGH-GI: 1450 ± 210 min, LCHF: 1460 ± 183 min, *p* = 0.469) and interval (LOW-GI: 657 ± 122 min, HIGH-GI: 622 ± 120 min, LCHF: 641 ± 85 min, *p* = 0.731) sessions.

### Body Composition

Body mass, BMI, and fat mass did not differ between groups before the intervention and were significantly reduced as a result of the intervention in all groups, as shown in Table 3. Based on the results of the BIA measurement, the LOW-GI (− 3.6 ± 3.5 kg, − 1.1 ± 1.0 kg m^−2^) and LCHF (− 4.0 ± 4.1 kg, − 1.3 ± 1.2 kg m^−2^) record greater losses in weight and BMI compared to HIGH-GI group (− 1.3 ± 2.2 kg, − 0.4 ± 0.6 kg m^−2^, *p* = 0.015 and *p* = 0.013, ηp^2^ = 0.119 and ηp^2^ = 0.124). Similarly, the changes in fat mass were greater in LOW-GI (− 3.8 ± 2.9 kg) and LCHF (− 3.9 ± 3.4 kg) compared to HIGH-GI (− 1.0 ± 2.0 kg, *p* = 0.002, ηp^2^ = 0.186). Fat-free mass did not change significantly in any of the groups (LOW-GI: + 0.1 ± 1.6 kg, HIGH-GI: − 0.4 ± 1.5 kg, LCHF: − 0.2 ± 1.5 kg). No significant differences were observed when comparing weight, BMI or fat mass between LOW-GI and LCHF group.

**Table 3:** Results from body weight and composition

Group	T-0	T-10	Time x Group	Time effect	Group effect at T-10
Body mass [kg]					
LOW-GI	79.5 ± 9.8	75.9 ± 8.1	**0.026**	** < 0.001**	0.799
HIGH-GI	77.1 ± 11.3	75.8 ± 9.8		**0.013**	
LCHF	81.5 ± 10.8	77.4 ± 8.8		** < 0.001**	
BMI [kg m^−2^]					
LOW-GI	24.1 ± 2.8	23.0 ± 2.2	**0.019**	** < 0.001**	0.876
HIGH-GI	23.7 ± 3.2	23.3 ± 2.8		**0.008**	
LCHF	24.6 ± 3.3	23.4 ± 2.7		** < 0.001**	
Fat mass [kg]					
LOW-GI	16.6 ± 6.0	12.8 ± 4.6	**0.002**	** < 0.001**	0.870
HIGH-GI	14.2 ± 7.4	13.2 ± 5.7		**0.043**	
LCHF	16.2 ± 8.2	12.3 ± 6.2		** < 0.001**	
Fat-free mass [kg]					
LOW-GI	63.0 ± 6.0	63.1 ± 5.8	0.558	0.530 *	0.317 *
HIGH-GI	62.9 ± 5.5	62.6 ± 5.2			
LCHF	65.3 ± 5.7	65.1 ± 6.1			

*Indicates main group effect from two-way mixed ANOVA. Bold numbers respresent a significant interaction effect or a significant simple main effect

### Metabolic Outcomes

**Fig. 3:**
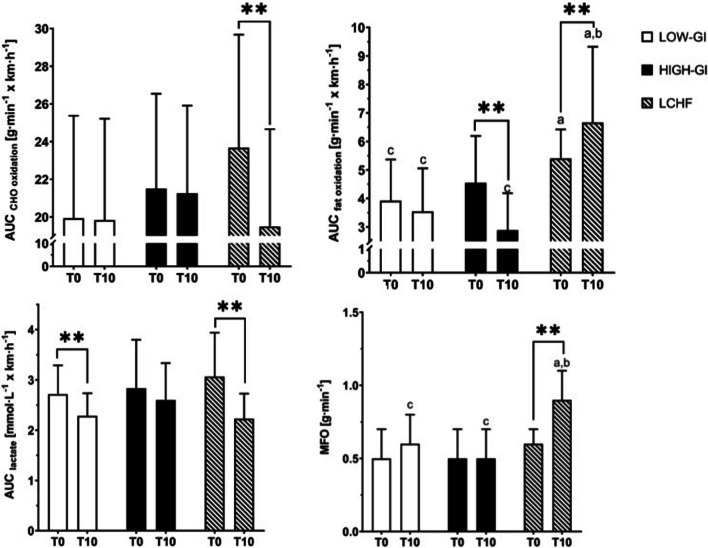
Summary of important metabolic outcomes. *, **indicate significant difference between T-0 and T-10, *p < 0.05, **p < 0.001. **a** indicates significant difference at T-10 compared to LOW-GI. **b** indicates significant difference at T-10 compared to HIGH-GI. **c** indicates significant difference at T-10 compared to LCHF. AUC, Area under the Curve; MFO, Maximum fat oxidation

No baseline differences were observed between groups in AUC RER, AUC blood lactate or for the values at exhaustion (*p* > 0.050, respectively). The AUC of the RER during the graded exercise test decreased significantly (− 0.04 ± 0.05; *p* < 0.001, d = 0.814) in the LCHF group. The simple effect of group at the final examination revealed a significantly lower AUC of the RER in LCHF compared to both carbohydrate groups (*p* = 0.002, ηp^2^ = 0.185). After 10 weeks, the participants of all groups had significantly lower RER values at exhaustion compared to baseline (*p* < 0.050, Table 4). The rate of change in RER at maximum effort differed statistically significant between the LCHF and carbohydrate groups (*p* < 0.001, ηp^2^ = 0.265). Participants in LOW-GI (− 0.4 ± 0.5 mmol L^−1^ × km h^−1^, *p* < 0.001, d = 0.888) and in the LCHF (− 0.8 ± 0.7 mmol L^−1^ × km h^−1^, *p* < 0.001, d = 1.199) had a statistically significant decrease in AUC of blood lactate concentrations during the graded exercise test (Fig. 3). Blood lactate concentrations at exhaustion were significantly lower in LCHF after the intervention (− 1.7 ± 1.9 mmol L^−1^, *p* < 0.001, d = 0.869) and differed significantly from the HIGH-GI group (*p* = 0.043, ηp^2^ = 0.096). No differences were found between the LOW-GI and LCHF group for the blood lactate concentration at exhaustion (Table 4).

**Table 4:** Results from metabolic outcomes

group	T-0	T-10	Time x Group	Time effect	Group effect at T-10
AUC RER					
LOW-GI	0.61 ± 0.04	0.59 ± 0.04^c^	**0.026**	0.122	**0.002**
HIGH-GI	0.61 ± 0.04	0.61 ± 0.04^c^		0.915	
LCHF	0.59 ± 0.04	0.55 ± 0.06^a,b^		** < 0.001**	
RER at exhaustion					
LOW-GI	1.18 ± 0.06	1.13 ± 0.046^c^	** < 0.001**	** < 0.001**	** < 0.001**
HIGH-GI	1.17 ± 0.048	1.14 ± 0.05^c^		**0.038**	
LCHF	1.18 ± 0.04	1.07 ± 0.05^a,b^		** < 0.001**	
Lactate concentration at exhaustion [mmol L^−1^]					
LOW-GI	9.5 ± 2.2	9.0 ± 1.9	**0.009**	0.161	**0.043**
HIGH-GI	9.9 ± 2.7	10.0 ± 2.1^c^		0.798	
LCHF	8.6 ± 1.2	8.4 ± 1.9^b^		** < 0.001**	
FAT_max_ [% VO_2 peak_]					
LOW-GI	59 ± 9	62 ± 10	0.617	0.025 *	0.089*
HIGH-GI	57 ± 12	59 ± 9			
LCHF	61 ± 11	67 ± 10			

^a,b,c^Significant different between groups at T-10: ^a^compared to LOW-GI, ^b^compared to HIGH-GI, ^c^compared to LCHF. *Indicates main group effect from two-way mixed ANOVA. Bold numbers respresent a significant interaction effect or a significant simple main effect

For AUC of fat and carbohydrate oxidation significant time group interactions were found (Fig. 3)**.** Before the intervention, AUC of fat oxidation was significantly lower in LOW-GI (2.6 ± 0.1 g min^−1^ × km h^−1^) compared to LCHF (3.6 ± 0.7 g min^−1^ × km h^−1^, *p* = 0.003, ηp^2^ = 0.171). No baseline differences for AUC of carbohydrate oxidation, maximum fat oxidation (MFO) and intensity at MFO (FAT_max_) were observed. Fat oxidation during the incremental exercise test remained unchanged in LOW-GI (− 0.3 ± 1.0 g min^−1^ × km h^−1^, *p* = 0.240), decreased significantly in HIGH-GI (− 1.1 ± 1.0 g min^−1^ × km h^−1^, *p* < 0.001, d = 1.113) and increased in LCHF (+ 0.8 ± 1.6 g min^−1^ × km h^−1^, *p* = 0.027, d = 0.522). After the intervention, AUC of fat oxidation was significantly higher in LCHF compared to both carbohydrate groups (*p* < 0.001, ηp^2^ = 0.429). AUC of carbohydrate oxidation remained unchanged in LOW-GI (− 0.1 ± 3.4 g min^−1^ × km h^−1^, *p* = 0.923) and HIGH-GI (− 0.2 ± 2.4 g min^−1^ × km h^−1^, *p* = 0.769) and decreased significantly in LCHF (− 2.8 ± 4.6 g min^−1^ × km h^−1^, *p* = 0.012, d = 0.601). In terms of MFO and FAT_max_ statistical analysis revealed a significant increase in MFO in LCHF (+ 0.4 ± 0.2 g min^−1^, *p* < 0.001, d = 1.605) while in the carbohydrate groups no changes were observed. MFO was significantly higher in LCHF (0.9 ± 0.2 g min^−1^) compared to LOW-GI (0.6 ± 0.2 g min^−1^) and HIGH-GI (0.5 ± 0.2 g min^−1^, *p* < 0.001, ηp^2^ = 0.489) after the intervention (Fig. 3). FAT_max_ showed no different change or time x group interaction (*p* > 0.05). Nevertheless, FAT_max_ showed an improvement over time regardless of the intervention group (+ 3 ± 12% VO_2 peak_, *p* = 0.025, ηp^2^ = 0.079, Table 4).

### Endurance Performance

**Fig. 4:**
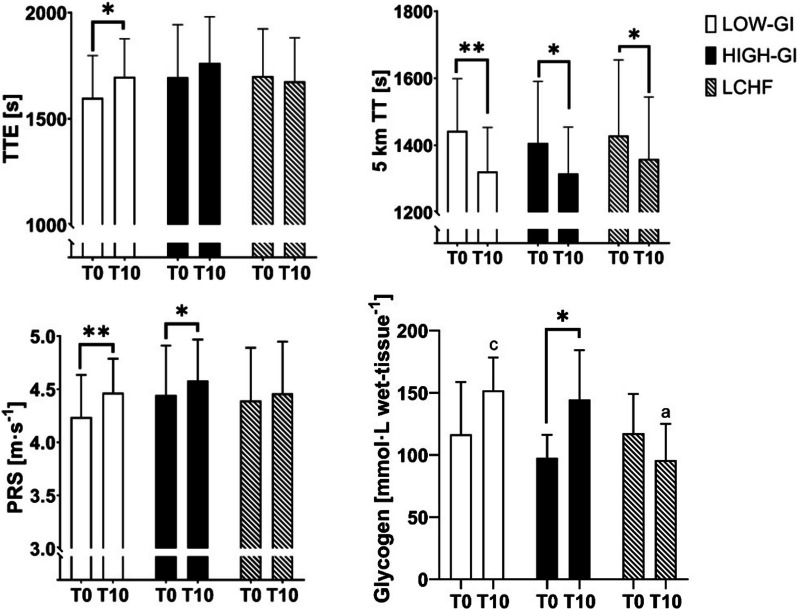
Summary of outcomes for endurance performance. *, ** indicate significant difference between T-0 and T-10, *p < 0.05, **p < 0.001. **a** indicates significant difference at T-10 compared to LOW-GI. **c** indicates significant difference at T-10 compared to LCHF. TTE, Time to Exhaustion; TT, Time Trial; PRS, Peak running speed

Significantly changed parameters in endurance performance are shown in Fig. 4. Baseline values were comparable between groups (*p* > 0.050). The duration of the graded exercise test, hereinafter always referred to as time to exhaustion (TTE), increased significantly in the LOW-GI (T0: 1598 ± 199 s, T10: 1698 ± 179 s, *p* = 0.005, d = 0.638). Although the simple main effect of time showed a strong trend for an improvement in HIGH-GI (T0: 1696 ± 248 s, T10: 1763 ± 217 s), the comparison between pre and post intervention nearly missed significance (*p* = 0.050, d = 0.467). TTE remained unchanged in LCHF (T0: 1700 ± 223 s, T10: 1677 ± 205 s, *p* = 0.498) group. The rate of change showed significantly significant differences between the LOW-GI (1.1 ± 0.9%) and LCHF group (1.0 ± 0.9%, *p* = 0.027, ηp^2^ = 0.110). Similar results also apply to the peak running speed (PRS) in the graded exercise test. PRS increased significantly in LOW-GI (+ 0.2 ± 0.2 m s^−1^, *p* < 0.001, d = 0.998) and HIGH-GI (+ 0.1 ± 0.2 m s^−1^, *p* = 0.017, d = 0.582) and remained unchanged in LCHF (+ 0.1 ± 0.2 m s^−1^, *p* = 0.078). Significant differences in improvements were found when comparing LCHF with LOW-GI group (*p* = 0.046, ηp^2^ = 0.095).

Unlike TTE and PRS, VO_2_ peak did not show any significant changes in the time course of the study (*p* > 0.050 for time*group interaction and main effects). Similar results were found for the 5-km time trial (TT). Participants in all groups were able to enhance time compared to the beginning of the intervention (main effect of time: *p* < 0.001, ηp^2^ = 0.550). The rate of change showed no significant difference between the groups (*p* = 0.189). However, it should not go unmentioned, that the highest improvement was found in the LOW-GI (− 121 ± 65 s, *p* < 0.001, d = 1.862), followed by the HIGH-GI (− 91 ± 120 s, *p* = 0.003, d = 0.753) and the LCHF (− 70 ± 96 s, *p* = 0.003, d = 0.724) group. No simple group effects were found before or after the intervention. In line with these results, no significant time*group interaction was found for running economy at lactate threshold (*p* = 0.444). Moreover, no different changes were observed between the groups (LOW-GI: T-0: 224 ± 27 vs. T-10: 220 ± 30 mL min^−1^ km^−1^, HIGH-GI: T-0: 228 ± 27 vs. T-10: 217 ± 24 mL min^−1^ km^−1^, LCHF: T-0: 214 ± 35 vs. T-10: 214 ± 25 mL min^−1^ km^−1^).

### Muscle Glycogen Content

Of the 24 subjects examined by magnetic resonance imaging at the beginning of the study, only 7 participants were analysed in the LOW-GI, 5 participants in the HIGH-GI, and 6 participants in the LCHF group at the end of the intervention, since the other subjects were dropouts. As shown in Fig. 4, the muscle glycogen content remained unchanged in the LOW-GI group (+ 35.4 ± 56.9 mmol L wet-tissue^−1^, *p* = 0.151) and increased significantly for participant of the HIGH-GI group (+ 42.6 ± 28.0 mmol L wet-tissue^−1^, *p* = 0.027, d = 1.522). In the LCHF group the glycogen content was reduced by − 21.5 ± 32.6 mmol L wet-tissue^−1^, but the reduction was not significant (*p* = 0.166). ANOVA revealed a significantly higher rate of change in the LCHF group compared to the HIGH-GI group (*p* = 0.043, ηp^2^ = 0.342). The glycogen content after the intervention was significantly lower in LCHF compared to LOW-GI (151.9 ± 26.5 vs. 95.8 ± 29.3 mmol L wet-tissue^−1^, *p* = 0.014, ηp^2^ = 0.435).

Two-way mixed ANOVA showed no significant interaction for intramyocellular lipids (*p* = 0.093). Main effect of time showed a significant increase in IMCL (T-0: 0.39 ± 0.19% of water signal, T-10: 0.43 ± 0.15% of water signal, *p* = 0.001, ηp^2^ = 0.521) independent of the nutritional intervention. The rate of change for the IMCL during the intervention did not differ significantly between the groups (*p* = 0.093).

## Discussion

The main objective of the study was to investigate whether a 10-week free-living endurance training in combination with a high-carbohydrate low glycaemic index diet could enhance parameters of fat metabolism to a similar extent as a low-carbohydrate diet (LCHF) without reducing maximum performance capacity in comparison to a high GI diet. After the low GI diet, statistically significant reductions in blood lactate concentration in the graded exercise test, as well as significant increases in peak running speed (PRS), time to exhaustion (TTE), and performance in the 5 km time trial (TT) was observed. Compared to baseline values neither fat oxidation, nor carbohydrate oxidation in the graded exercise test showed any changes after the low GI diet. Muscle glycogen content did not change significantly following the LOW-GI group, but was significantly higher after the intervention compared to the LCHF group. In comparison, although relevant changes in metabolism were observed in the LCHF group in the form of reduced blood lactate concentration, increased AUC of fat oxidation and MFO during the graded exercise test, no improvements were observed in peak running speed and TTE. Nevertheless, an improvement in the 5 km TT was also observed in the LCHF group. Albeit, the strength of the effect of the LCHF diet (d = 0.724) on 5 km TT performance was smaller compared to the LOW-GI group (d = 1.862). After a HIGH-GI diet, fat oxidation during the exercise test decreased compared to baseline measurement. However, peak running speed in the graded exercise test and glycogen stores were significantly increased compared to the beginning of the study. Although not significant TTE showed a strong trend for improvement in HIGH-GI group.

In line with results from other LCHF-investigation [21, 45–47], measured fat oxidation in this study increased in LCHF group, remained unchanged in LOW-GI, and decreased during the HIGH-GI diet. Fat oxidation after the intervention was significantly higher in LCHF compared to both carbohydrate groups. Carbohydrate oxidation showed no changes in the LOW- or HIGH-GI groups, but decreased significantly in LCHF group. Metabolic changes during a long-term (> 2 weeks) LCHF diet result in increased availability of free fatty acids and reduced carbohydrate oxidation [48]. The reciprocal relationship between carbohydrate and fat oxidation is a consequence of altered acetyl-CoA supply from glycolysis or beta oxidation or a possible modified conversion of pyruvate to acetyl-CoA and hence a more efficient coupling of glycolysis with Krebs cycle [15, 49]. Insulin, an anabolic hormone, serves as a key regulator in substrate oxidation and high amounts of circulating insulin decrease fatty acid oxidation via several pathways [50–52]. As a consequence, a low-carbohydrate diet with constantly lower insulin levels, may result in enhanced oxidation of fatty acids [52, 53], while a high-carbohydrate diet with higher insulin levels can have the opposite effect on fat oxidation [13]. It is moreover noteworthy, that we observed an unchanged fat oxidation in LOW-GI group. However, because fat oxidation can only be calculated until RER = 1.00, lactate concentration during the incremental test should not be disregarded since it might be able to mirror all steps of the graded exercise test. Evidence suggests, that there is a link between lower blood lactate concentration and higher fat oxidation rates during exercise [54, 55]. Therefore, although we could not observe a significant increase in fat oxidation in the LOW-GI group, the significant decrease in the AUC of the lactate concentration could be interpreted as an indirect indicator, suggesting that the adherence to a low GI diet, and thereby reduced insulin levels, may have improved fat oxidation during the graded exercise test [27]. However, to date, there are only few studies investigating the longer-term effects (> 2 weeks) of a variable carbohydrate diet on substrate metabolism. The results of the pilot trial by Zdzieblik, Friesenborg [31] including 28 males and a 4-week diet intervention, are in line with the present study. The working group assessed fat oxidation based on changes in blood lactate concentration and observed significant improvements in the LCHF and low GI (39 ± 4) groups. No differences in performance at first threshold were found in a 3-week nutritional intervention with 12 male and 8 female runners when a low glycaemic index diet (39 ± 1) was compared to a moderate GI diet (61 ± 1) [56]. In contrast, another study by Durkalec-Michalski, Zawieja [57], where 10 males and 7 females followed a 3-week low or moderate glycaemic index diet, was unable to show differences in substrate metabolism during an incremental cycle test between the two different glycaemic indices groups. One possible explanation might be, that the duration of the study of 3 weeks was not sufficient for substrate metabolism to adapt to the small difference between moderate and low glycaemic index. As shown in the observation of Hamzah, Higgins [58], where no differences in substrate oxidation or running performance after five days of high (71 ± 1) or low glycaemic (36 ± 0) index diets were profound. Thus, the benefits of a low glycaemic index diet and associated low postprandial insulin levels are likely to depend on the duration of the intervention.

In the present 10-week intervention, improvements in performance in terms of time to exhaustion (TTE) and peak running speed in the graded exercise test were profound in the LOW- and HIGH-GI group, whereas no differences were observed in the LCHF group. The performance in the 5 km TT improved regardless of the nutritional intervention. Additionally, VO_2_ peak or running economy did not show any significant changes in the time course of the study as also observed in other studies [59, 60]. However, in an observation with elite race walkers, a LCHF diet led to an impairment in exercise economy, but we were not able to see this in our data [25, 61]. Nonetheless, these results are in accordance with the observations in the pilot trial by Zdzieblik, Friesenborg [31], where the TTE of an incremental cycling test improved in the two carbohydrate groups, which had varying glycaemic indices, but it significantly declined in the LCHF group. After the 4-week intervention, VO_2_ peak remained unchanged in all three experimental groups and the absolute maximum cycling performance improved only in the group with the high glycaemic index diet (74 ± 3). Relative peak power output improved significantly more in the low GI group than in the LCHF group [30]. Further supporting data comes from Durkalec-Michalski, Zawieja [56]. In a 3-week nutritional intervention, a low glycaemic index diet (39 ± 1) resulted in a slight improvement in TTE and running performance compared to a moderate glycaemic index diet (61 ± 1).

While only the LCHF group showed an improvement in fat oxidation combined with a decreased carbohydrate oxidation and the HIGH-GI group increased peak running speed and showed a trend towards an improvement in TTE (*p* = 0.050), it appears that the LOW-GI group might combine the effects on substrate metabolism and performance. Still, subjects in the LOW-GI group showed no changes in fat oxidation, but compared to baseline a lower lactate concentration during the graded exercise test on the treadmill, which might be indicative for an increased fat oxidation [55]. Further, an improved peak running speed and TTE were observed in the LOW-GI group. The more efficient available substrates (glycogen, triglyceride, plasma glucose and plasma fatty acids) are used to cover increased energy requirements during exercise, the greater the metabolic flexibility [62]. The lower lactate concentration, PRS and TTE in the graded exercise running test, as well as the increased performance in the 5 km TT and the unchanged muscle glycogen, suggest an improved metabolic flexibility in the LOW-GI group. In the LCHF group, due to the decrease in carbohydrate oxidation and the limited PRS in the graded exercise test, it can be assumed that as a result metabolic flexibility is impaired possible due to reduced glycogenolysis and pyruvate dehydrogenase activity, the two major pathways of carbohydrate breakdown [63]. While the high GI diet showed significant improvements in PRS and TT performance, a decrease in fat oxidation was observed.

Muscle glycogen stores increased in the HIGH-GI group, remained unchanged in LOW-GI and LCHF, however, after 10 weeks glycogen content was lower in LCHF group compared to LOW-GI group. Glycogen stores were measured using magnetic resonance spectroscopy, which is a reliable non-invasive alternative to muscle biopsy [64, 65]. During exercise, muscle glycogen is the main carbohydrate substrate and therefore crucial for maintaining muscle contraction during prolonged endurance exercise [11, 66, 67]. After exercise, the level of glucose and insulin play an essential role in the recovery of glycogen. After carbohydrate ingestion, when glucose availability and insulin are high, the rate of glycogen synthesis and accumulation increases [66, 68]. Consequently, consuming high GI carbohydrates after glycogen-depleting exercise promotes glycogen restoration [69], which might be a possible explanation for the increase in muscle glycogen content in the HIGH-GI group. A meta-analysis showed that glycogen content is directly affected by carbohydrate availability and fitness status [70]. The drastically reduced carbohydrate intake in the LCHF group resulted in significantly lower muscle glycogen content compared to LOW-GI group, which, despite improved fat oxidation, may have been one of the performance limiting factors in the graded exercise test. In the LOW-GI and HIGH-GI group, carbohydrate intake led to a maintenance or increase in glycogen stores and thus to an improvement in high intensity endurance performance. Furthermore, intramyocellular lipids (IMCL), which serve as a fuel for fat oxidation in mitochondria [71], increased in all groups equally. Endurance training leads to an increase in lipoprotein lipase activity [72], which has a direct influence on the accumulation of IMCL [73]. Despite other observations [74–76], the fat content of the daily diet did not influence IMCL content in the present investigation. Nonetheless, sample size for MRS measurements was small (n = 18). It could be speculated that IMCL increased as a consequence of endurance training. It is possible that a LCHF diet and the associated higher IMCL content contribute to improved fatty acid oxidation during exercise, but exact regulatory mechanisms are not yet fully understood and need to be further investigated [77].

In the current investigation favourable changes in body composition in recreationally active men have been observed in all groups. However, following a low GI or LCHF diet, reductions in body mass and fat mass were significantly greater compared to a high GI diet. It is not assumed that these results can be attributed to differences in energy intake between the low GI and LCHF diet, as the mean energy intake was not significantly different during the intervention between those groups. Further, the changes in body mass and fat were also significant in the HIGH-GI group, which excludes the possibility that reduced energy intake is responsible for the weight loss. Yet, in the LOW-GI group energy intake was reduced over time. Compared to data from Heatherly, L.G. [78], where a reduction in energy intake by approximately 1000 kcal per day during a 3 week ketogenic diet led to a 3% decrease in body mass, our data suggest a greater weight loss (around 5%) with only roughly 200 kcal reduction in daily energy intake. Nonetheless, the varying duration of the studies must also be taken into account. A desirable adjustment in body composition in endurance sports is one in which fat mass is reduced while fat-free mass is preserved. In recent years a low-carb diet has often been suggested for this purpose [79]. Our data suggest that the low GI diet was also capable of achieving these adaptations to a similar degree. How the glycaemic index affects body composition in endurance athletes has been studied sparsely so far. However, both Zdzieblik, Friesenborg [31] and Durkalec-Michalski, Zawieja [56] have shown that the glycaemic index might be important in improving body composition. From a physiological perspective, increased fat oxidation at rest and low insulin levels are, among other factors, likely to be responsible.

Daily energy intake decreased in the LCHF and LOW-GI groups, although only the decrease in the LOW-GI group was significant, the changes in the LCHF group just missed significance (*p* = 0.053). In both groups, protein intake also increased relative to total energy intake. It has been shown that increased protein intake promoted feelings of fullness and satiety [80, 81]. Furthermore, a large 3-year randomized trial demonstrated that a high protein, low GI diet can suppress hunger in overweight individuals [82]. The high fibre content coupled with the consistent insulin levels of the low GI diet may likely result in longer-lasting satiety [83] and therefore in a decreased energy intake. Additionally, some studies suggest that a LCHF diet may reduce objective hunger as measured by total ghrelin and glucagon-like peptide 1 (GLP-1) in endurance-trained individuals [84, 85].. It should also be noted that absolute carbohydrate intake decreased significantly in the LOW-GI group compared to the intake before the intervention. However, since energy intake also decreased significantly, it makes sense to consider carbohydrate intake in relation to energy intake. Yet, in this case, the carbohydrates content of approximately 50% was within the recommended amount for recreational athletes [86, 87]. If the training plan had been designed for competitive athletes, one would have had to pay attention to a higher carbohydrate intake. Nonetheless, there is some body of evidence that nutrition knowledge among non-elite athletes is poor and current recommendations regarding carbohydrate intake are only achieved by a very small percentage [88–91]. Apart from that, the LOW-GI group was very well able to implement the recommendations with respect to the glycaemic index. We are aware that when calculating the glycaemic index of a meal, there can be significant variations, but it has already been shown that if the same method is used, this error becomes negligible [92, 93]. On the other hand, the group with the high glycaemic index diet was barely able to reach the GI > 70 on average during the study. Excessive fluctuations in blood glucose concentration cause a higher feeling of hunger, and thus an increased energy intake. Because of this, the long-term diet with a high glycaemic index may have been more difficult to implement.

### Limitations

This trial also has some limitations. Diet for the study period was monitored using self-reported 24-h recalls, which may be susceptible to reporting bias, recall bias and training bias [94]. 24 h recalls were completed two times per week, reducing the likelihood of random errors, and furthermore, this method is less likely to be affected by underreporting than other methods, such as food records [95]. It is also known that the first recall could be a significant point of bias [96]. To minimise this error, an additional food frequency questionnaire was used to assess diet before the study [97].

Although previously assumed, we did not observe any differences between the diets during the 5 km TT. A possible reason for this could be that compared to cycling more muscle mass is addressed during running and less glycogen is broken down in the leg muscles and the m. gastrocnemius is not depleted for glycogen at exhaustion [98]. It might be anticipated that the differences in muscular glycogen stores will become more pronounced during shorter more intense bouts of exercise. However, the groups with the higher glycogen stores showed higher effects on improvement in performance at 5 km TT.

Furthermore, future investigations should include different sex groups and use different periodisation of macronutrient intake to better understand underlying mechanism. The analysis with metabolomics methods could further shed light on the ongoing adaptions in metabolism.

## Conclusions

To the best of our knowledge, this is the first study to examine the effects of diets that differ in carbohydrate content and glycaemic index over a period of 10 weeks under free-living conditions. In conclusion, besides a decreased energy intake, the effects of a low GI diet were a decrease in blood lactate concentrations during exercise, an improvement in 5 km TT and maximum values at the graded exercise test—PRS and TTE—and the maintenance of glycogen stores. By comparison LCHF diet resulted in an improved fat oxidation in the incremental test while altering carbohydrate oxidation, training adaptions at higher intensities and muscle glycogen restoration due to lack of carbohydrate provision. Although a high GI diet resulted in improved performance at high intensities and increased muscle glycogen content, fat oxidation was impaired after 10 weeks. Despite the positive effects on body composition and fat oxidation, a low-carb diet might be negatively affecting long-term health, because of the high fat content and a decrease in the intake of essential macronutrients [99] and should only be recommended with caution. In the LOW-GI group, the facilitated utilisation of fats due to decreased plasma lactate concentrations combined with unaffected carbohydrate metabolism during higher intensities could led to result in improved metabolic flexibility. In spite of the significantly increased muscle glycogen content, changes in metabolism in the HIGH-GI group might impair the ability to shift from carbohydrate to fat oxidation in response to different exercise intensities. Taking these findings together, the present data suggest that the long-term implementation of a low GI diet results in favourable changes in substrate oxidation during prolonged exercise and improved endurance performance, in contrast to a LCHF or high GI diet.

### Supplementary Information

**Additional file 1. Table 1.** Nutritional guidelines for each intervention group. **Table 2**. Example meal plan for one day for every intervention group. **Table 3**. Example week of the prescribed endurance training plan.

## Data Availability

The datasets used and analysed during the current study are available from the corresponding author on reasonable request.

## Abbreviations

ATP: Adenosine triphosphate
AUC: Area under the curve
GI: Glycaemic index
GLP: Glucagon-like peptide 1
IMCL: Intramyocellular lipid
LCHF: Low carbohydrate high fat
MRS: Magnetic resonance spectroscopy
PRS: Peak running speed
RER: Respiratory exchange ratio
TT: Time trial
TTE: Time to exhaustion
