# Supplementary material for: Effects of a 10-Week Exercise and Nutritional Intervention with Variable Dietary Carbohydrates and Glycaemic Indices on Substrate Metabolism, Glycogen Storage, and Endurance Performance in Men: A Randomized Controlled Trial
Source: Sports Med Open. 2024 Apr 10;10:36. doi: 10.1186/s40798-024-00705-9 (PMC11006643; doi:10.1186/s40798-024-00705-9)
Supplement: Supplementary file 1 — Additional file 1. Table 1. Nutritional guidelines for each intervention group. Table 2. Example meal plan for one day for every intervention group. Table 3. Example week of the prescribed endurance training plan. [file 40798_2024_705_MOESM1_ESM.docx]

Sports Medicine Open

**Effects of a 10-week Exercise and Nutritional Intervention with variable dietary Carbohydrates and Glycaemic Indices on Substrate Metabolism, Glycogen Storage, and Endurance Performance in Men: A Randomized Controlled Trial**

Anna Maria Moitzi ^1,2,3*^, Martin Krššák ^4,5^, Radka Klepochova ^4,5^, Christoph Triska ^6,7^, Robert Csapo ^7^ and Daniel König ^1,3^

^1^ Division of Nutrition, Exercise and Health, Department of Nutritional Sciences, Faculty of Life Sciences, University of Vienna, Vienna, Austria.

^2^ Vienna Doctoral School of Pharmaceutical, Nutritional and Sport Sciences, University of Vienna, Vienna, Austria.

^3^ Division of Exercise and Health, Department of Sport and Human Movement Science, University of Vienna, Vienna, Austria.

^4^ High field MR Centre of Excellence, Department of Biomedical Imaging and Image guided Therapy, Medical University of Vienna, Vienna, Austria.

^5^ Division of Endocrinology and Metabolism, Department of Internal Medicine III, Medical University of Vienna, Vienna, Austria.

^6^ Leistungssport Austria, High Performance Centre, Brunn am Gebirge, Lower Austria, Austria.

^7^ Division of Training Science, Department of Sport and Human Movement Science, University of Vienna, Vienna, Austria.

* Correspondence: [anna.moitzi@univie.ac.at](mailto:anna.moitzi@univie.ac.at)

# Supplementary Material

Table 1 Nutritional guidelines for each intervention group.

|  | **HIGH-GI** | **LOW-GI** | **LCHF** |
| --- | --- | --- | --- |
| **Carbohydrate intake** | 50-60% (65% of CHO with GI > 70) | 50-60% (65% of CHO with GI < 50) | ≤ 50 g per day |
| **Fat intake** | 25% | 25% | ≥ 65% |
| **Protein intake** | 15 – 20 % | 15 – 20% | 15 – 20% |
| **Meat, fish and eggs** | no restrictions, but in small quantities and in low fat version  *Examples:* steak, salmon, … | no restrictions, but in small quantities and in low fat version  *Examples:* steak, salmon, … | no restriction except for breaded products  *Examples:* steak, salmon, … |
| **Vegetables** | only as side dish, but no as main source of carbohydrate | no restriction except for potatoes | low-starch vegetables such as cabbage, cucumber, lettuce and tomatoes |
| **Fruits** | dried dates and sweet fruits | no restrictions expect for dates, bananas and sugared fruits | a maximum of 200g of berries |
| **Dairy products** | sweetened dairy products like yoghurts or rice drinks, low fat version should be preferred | low-fat cheese or yoghurt, cottage cheese and buttermilk, but no flavoured industrial dairy products | whole milk yoghurt and cream cheese |
| **Cereal products** | white flour products  Examples: toast, white rice, couscous, bulgur, … | wholemeal products  Examples: oats, wholemeal bread, brown rice, … | not allowed |
| **Nuts and seeds** | no restrictions | no restrictions | no restrictions |
| **Beverages** | no restrictions | unsweetened teas, water and sugar-free lemonades | unsweetened teas, water and sugar-free lemonades |
| **Sweeteners, candies and snacks** | Chocolate, rice cake, … | dark chocolate (≥ 70% cocoa), sugar-free jam or cinnamon | dark chocolate (≥ 70% cocoa) or cinnamon |

Table 2 Example meal plan for one day for every intervention group.

|  | **HIGH-GI** | **LOW-GI** | **LCHF** |
| --- | --- | --- | --- |
| **Breakfast** | Pancakes with different toppings:   - maple syrup - jam - banana | Oats with milk and berries | Omelette from eggs with tomatoes and champignons |
| **Lunch** | Chili con carne with a bun | Brown rice with vegetables and chicken breast | Coconut soup made from coconut milk with seafood, courgette, belly pepper, curry and other spices as required |
| **Dinner** | Chicken breast with mashed potatoes | Gazpacho | Meat with vegetables and crème fraiche |
| **Snack** | dried dates | fruits and nuts | berries and nuts |

Table 3 Example week of the prescribed endurance training plan.

| **T – 8 weeks** |  | **Training week 3** |
| --- | --- | --- |
| **Training** | **Intensity** | **Duration (min)** |
| Constant load run | GA1 | 50 |
| Interval session | 10:00 Warm-up  1:30 GA2 + 1:00 rest  3x(0:30 WSA + 1:00 rest)  1:30 GA2 + 1:00 rest  3x(0:30 WSA + 1:00 rest)  10:00 Cool down | 35 |
| Constant load run | REG | 40 |
| Interval session | 5:00 Warm-Up  20x(1:00 GA1 + 1:00 REG) | 25 |
| Constant load run (long run) | GA1 – GA1-2 | 100 |
